# Ornithine decarboxylase influences granulosa cell proliferation and steroidogenesis: implications for ovarian regression in Wuding chickens

**DOI:** 10.3389/fvets.2026.1801162

**Published:** 2026-04-20

**Authors:** Xinyu Ma, Wei Zhu, Chen Li, Qiang Zhang, Xinyang Fan, Yongwang Miao

**Affiliations:** 1 College of Animal Science and Technology, Yunnan Agricultural University, Kunming, Yunnan, China; 2 Institute of Animal Genetics and Breeding, Yunnan Agricultural University, Kunming, Yunnan, China; 3 Yunnan Yunling Guangda Yukou Poultry Industry Co., Ltd., Kaiyuan, Yunnan, China

**Keywords:** broodiness, cell proliferation, c-MYC signaling axis, granulosa cells, ornithine decarboxylase (ODC), steroidogenesis, Wuding chicken

## Abstract

Follicular development critically determines egg-laying efficiency in poultry, a process precisely regulated by the equilibrium of granulosa cell (GC) proliferation, apoptosis, and steroidogenesis. Ornithine decarboxylase (ODC), the rate-limiting enzyme in polyamine biosynthesis, is a central regulator of cell proliferation and embryonic development. In this study, Wuding chickens, a breed exhibiting pronounced broodiness, were employed to elucidate ODC’s role in follicular maturation and the transition from laying to broody state. Comprehensive analysis of *ODC* expression across the hypothalamic–pituitary-gonadal (HPG) axis revealed significantly elevated levels in ovaries, pituitaries, and hypothalami of laying hens compared to broody hens. Primary GC cultures derived from Wuding chickens demonstrated that ODC promotes cell cycle progression via *c-MYC* upregulation and subsequent cyclin activation. Concurrently, ODC suppresses GC apoptosis by elevating *BCL2* and suppressing *Caspase-3*, while enhancing steroidogenic capacity through coordinated regulation of key genes (*FSHR*, *STAR*). Our *in vitro* data demonstrate that ODC modulates the expression of *SAT1* and *PAOX* genes, thereby maintaining elevated intracellular polyamine levels in GCs to sustain their proliferative activity. Collectively, ODC functions as a positive regulator of GC physiology in Wuding chickens, driving follicular development through dual mechanisms: c-MYC/cyclin-mediated proliferation and polyamine metabolic adaptation. These findings elucidate the metabolic and molecular mechanisms underlying the laying-to-broody transition in chickens.

## 1 Introduction

Egg production efficiency is a key economic trait in commercial poultry operations, significantly influencing farm profitability, feed conversion ratios, and sustainable production metrics. Although local chicken breeds possess superior egg quality, they generally suffer from low egg-laying rates and strong broodiness, which restrict industrial development. Broodiness, a conserved maternal instinct in birds, induces physiological changes such as ovarian atrophy, cessation of laying, and nesting behavior, leading to interruptions in the reproductive cycle and severely impairing laying efficiency (1). The Yunnan Wuding chicken (*Gallus gallus* domesticus) serves as a typical local breed model; hens typically enter a broody period lasting 6 ~ 20 days after laying 14 ~ 16 eggs. During this period, ovarian atrophy occurs, providing an irreplaceable natural model for analyzing the molecular mechanisms of follicular atresia and development (2).

The core regulatory unit of hierarchical follicular development is the “two-cell, two-gonadotropin” system comprising Granulosa cells (GCs) and theca cells (3). Theca cells, located in the outer follicular layer, synthesize androgens such as androstenedione under Luteinizing Hormone (LH) stimulation; these androgens serve as precursors for estrogen synthesis (4). GCs, located in the inner layer, act as the functional hub of follicular development. Under Follicle-Stimulating Hormone (FSH) stimulation, GCs synthesize aromatase (CYP19A1) to efficiently convert theca-derived androgens into estradiol (E2) and utilize cholesterol to synthesize progesterone (P4). Nie et al. reported in chicken GCs that co-treatment with FSH and WIF1 promotes cell proliferation and P4 secretion (5). In the late stages of follicular development (specifically the F1 follicle, the dominant preovulatory structure in the chicken ovary with a diameter of 30–40 mm), GCs express high levels of functional LH receptors to directly respond to LH signaling, thereby promoting P4 synthesis and ovulation (6). GCs also communicate directly with oocytes via gap junctions, providing nutrients and regulatory signals to support oocyte maturation (7). The proliferation and apoptosis of GCs directly determine the developmental fate of hierarchical follicles. High proliferative activity and low apoptosis rates in GCs promote sustained E2 and P4 secretion, driving continuous follicle maturation. Conversely, suppressed proliferation and increased apoptosis lead to atresia of hierarchical follicles and arrested development, thereby affecting egg-laying efficiency. Given the critical role of granulosa cells (GCs) in folliculogenesis, their molecular regulation of proliferation, apoptosis, and steroidogenesis is pivotal for deciphering follicular development mechanisms and enabling targeted molecular interventions to enhance avian egg-laying efficiency. GCs exhibit robust responses to FSH/LH stimulation and genetic perturbations, accurately recapitulating *in vivo* follicular physiology, thus establishing them as the optimal *in vitro* model for this study.

The polyamine metabolic pathway plays a critical role in reproductive regulation. Polyamines, primarily including Putrescine (Put), Spermidine (Spd), and Spermine (Spm), are positively charged low-molecular-weight organic compounds. Under physiological pH, they bind specifically to negatively charged macromolecules such as DNA, proteins, and phospholipids via electrostatic interactions, thereby precisely regulating key biological processes like cell proliferation, apoptosis, and embryonic development (8). Ornithine decarboxylase (ODC), the rate-limiting enzyme of polyamine biosynthesis, catalyzes the decarboxylation of L-ornithine to generate putrescine, and its activity is dynamically regulated by gonadotropins (LH/FSH) (9). In mouse models, putrescine supplementation increases serum levels of Gonadotropin-Releasing Hormone (GnRH) and P4 (10). Notably, ODC reduces miscarriage rates and the birth of malformed offspring in mice by ensuring correct chromosome segregation during oocyte meiosis (11). Fenelon et al. found in mink uterine cells that increased ODC activity promotes putrescine synthesis, thereby enhancing uterine cell proliferation and embryonic development (12). This indicates that ODC is associated with animal reproduction. In goose follicles, putrescine is positively correlated with E2, while spermine and spermidine are positively correlated with P4 (13). Spermidine regulates the transcription efficiency of Steroidogenic acute regulatory protein (STAR) by modulating the hypusination of the eukaryotic translation initiation factor 5A (eIF5A), thereby influencing steroid hormone secretion and regulating follicular development (14). This suggests that ODC may mediate important functions in steroid hormone synthesis via polyamine products.

Despite the increasing body of evidence supporting ODC’s critical role in mammalian reproductive regulation, its specific function and regulatory mechanisms in avian follicular development—particularly during the laying-to-brooding transition—remain poorly understood. In a preliminary transcriptomic analysis of Wuding chicken ovaries, we observed that *ODC* gene expression was significantly downregulated during the brooding period compared to the laying period (2). Based on this finding, we propose a mechanistic hypothesis: during the broody period in Wuding chickens, *ODC* downregulation inhibits polyamine synthesis, suppressing GC proliferation, activating apoptotic programs, and inhibiting steroid synthesis, ultimately triggering follicular atresia. Conversely, during the laying period, high *ODC* expression promotes polyamine synthesis, enhancing GC proliferation, inhibiting apoptosis, and synergistically promoting steroid synthesis to accelerate follicular development and improve egg-laying efficiency. Notably, prior studies have demonstrated that ODC promotes GC proliferation and suppresses apoptosis to regulate ovarian function in geese (15), yet the specific molecular mechanism by which ODC coordinates this physiological transition remains unexplored. This study employed Wuding chickens as a model to systematically characterize the spatiotemporal expression dynamics of *ODC* during the laying-brooding transition. Combined with gain-of-function and loss-of-function assays in primary GCs cultures, we elucidated the molecular mechanisms by which ODC regulates GC proliferation, apoptosis, and steroidogenesis. This study aims to bridge the gap in understanding the link between polyamine metabolism and steroid synthesis in avian reproduction, providing key targets for improving the egg-laying efficiency of local chicken breeds through molecular tools.

## 2 Materials and methods

### 2.1 Ethics statement

All experiments involving animals were conducted in accordance with the “Guidelines for the Care and Use of Laboratory Animals” and conformed to the guiding principles of the Animal Care and Use Committee of Yunnan Agricultural University (Approval No: APYNAU202503109).

### 2.2 Experimental animals and sample collection

Wuding chickens, a local breed from Wuding County, Yunnan Province, China, were selected for this study. This breed has not undergone intensive selection and retains complete broodiness traits, making it an ideal model for studying avian reproductive plasticity. Healthy Wuding chickens in the peak-laying phase with uniform body weight and consistent external morphology were selected from Wuding Shouyu Agricultural Development Co., Ltd. and housed individually under standardized conditions. Peak egg-laying was defined as the period when the flock-level egg-laying rate exceeded 80%. Based on egg-laying records and behavioral manifestations (e.g., cessation of laying, nesting, elevated body temperature), hens exhibiting typical characteristics were selected for each of the laying and broody groups. Hens were euthanized by cervical dislocation, and tissues including the heart, liver, spleen, lungs, kidneys, ovaries, pituitary gland, cerebellum, cerebrum and hypothalamus were immediately collected. Samples were rapidly placed in RNase-free cryotubes, preserved in liquid nitrogen, and transported to the laboratory for RNA extraction.

### 2.3 Gene cloning and bioinformatics analysis

Total RNA was isolated from tissues using TRIzol reagent, and RNA integrity was evaluated by 1.5% agarose gel electrophoresis, with assessment of the 28S/18S rRNA ratio. The purity and concentration of total RNA were measured using a Nano-400A spectrophotometer (ALL SHENG, Hangzhou, China). cDNA was synthesized using Oligo(dT)18 primers (500 μg/mL) and a reverse transcription kit (TaKaRa, Dalian, China). The synthesized cDNA was diluted to 300 ng/μL and stored at −20 °C for future use. Based on the chicken *ODC* mRNA sequence in the NCBI database (NM_001167766.2), primers were designed using Primer Premier 5 and Oligo 7 software (Supplementary Table S1) to amplify the coding sequence (CDS) of the Wuding chicken *ODC* gene. All primers were synthesized by Sangon Biotech (Shanghai) Co., Ltd. The PCR reaction system (10 μL) consisted of 0.4 μL each of forward and reverse primers (10 μmol/L), 5 μL of 2 × Es Taq Master Mix (CWBIO, Beijing, China), 1 μL of cDNA template (300 ng/μL), and 3.2 μL of ddH₂O. The PCR amplification program was as follows: pre-denaturation at 94 °C for 5 min; followed by 34 cycles of denaturation at 94 °C for 30 s, annealing at 58.5 °C for 30 s, and extension at 72 °C for 45 s; with a final extension at 72 °C for 5 min. Bioinformatics tools were used to analyze molecular characteristics, protein physicochemical properties, domains, and phylogenetic trees; the bioinformatics websites used are listed in Supplementary Table S2.

### 2.4 Gene expression analysis

Real-time quantitative PCR (RT-qPCR) was employed to detect the expression of relevant genes in different tissues and GCs of Wuding chickens. RT-qPCR was performed using a SYBR Green qPCR kit (dib), with GAPDH serving as the internal control for normalization. The PCR reaction system included: 0.8 μmol/L each of forward and reverse primers, 10 μL SYBR qPCR SuperMix Plus, 6.4 μL ddH₂O, and 2 μL cDNA template (200 ng/μL). The reaction program was carried out according to the manufacturer’s instructions. Specificity of the products was confirmed by melting curve analysis (single peak), and each sample was run in triplicate. RT-qPCR data were analyzed using the 2^−△△Ct^ method, and results were compared with the control group.

### 2.5 Isolation and culture of GCs

This study employed GCs isolated from preovulatory hierarchical follicles (F1–F3 stages; 8–40 mm diameter) of peak-laying Wuding hens as the *in vitro* model. These cells were rigorously isolated, characterized, and cryopreserved by our group to ensure experimental reproducibility. Cells were identified by positive expression of *FSHR*, *STAR*, and *CYP19A1* (see Methods in (1) for detailed protocols). Briefly, laying Wuding hens at peak production were selected from Wuding Shouyu Agricultural Co., Ltd. and sacrificed by cervical dislocation. Ovaries were harvested, and hierarchical follicles (F1–F3) were isolated. The yolk was drained after puncture, and the granulosa layer was peeled off. The granulosa tissue was minced into 1–3 mm fragments and digested in a mixture of 1 mg/mL Collagenase Type II and 0.25% Trypsin at 37 °C for 10 min. Digestion was terminated by adding complete medium (89% DMEM with 1.5 g/L NaHCO3, 10% fetal bovine serum, and 1% penicillin–streptomycin). The suspension was filtered through a 200-mesh sieve, and the filtrate was centrifuged at 1800 r/min for 10 min to collect the pellet. Cells were washed with complete medium to remove residual collagenase and debris, then centrifuged at 1800 r/min for 5 min, and the supernatant was discarded. Cells were resuspended in complete medium, seeded in culture dishes, and incubated at 37 °C with 5% CO_2_. After 3 h of differential attachment, the medium was replaced with fresh complete medium, which was subsequently changed every 24 h.

### 2.6 Construction of *ODC* overexpression vector, siRNA interference, and cell transfection

For the *ODC* overexpression experiment, primers targeting the *ODC* CDS were designed and synthesized (Supplementary Table S1) to amplify the CDS of the *ODC* gene, which was verified by Sanger sequencing. The target fragment was inserted into the pEGFP-N1 vector by double restriction enzyme digestion, and the specific restriction enzyme sites used for vector construction were designated SalI and KpnI. The recombinant vector was verified by Sanger sequencing to ensure no mutations in the inserted CDS sequence and correct insertion direction; it was named EGFP-ODC. Specific siRNA sequences were designed and synthesized by Sangon Biotech (Shanghai) Co., Ltd., including two experimental groups (si-523 and si-910) and a negative control group (NC); sequences are listed in Supplementary Table S3. When the GCs reached approximately 80% confluence, transfection was performed using Lipofectamine 2000 (Invitrogen, Carlsbad, USA) according to the manufacturer’s instructions. Plasmid vectors or siRNA were first diluted in 50 μL of serum-free Opti-MEM I reduced-serum medium (or other serum-free medium). Subsequently, an appropriate amount of Lipofectamine 2000 (gently vortexed before use) was diluted in 50 μL Opti-MEM I medium and incubated at room temperature for 5 min. Following incubation, the diluted Lipofectamine 2000 was mixed with the diluted plasmid or siRNA solution, gently vortexed, and further incubated at room temperature for 20 min to form transfection complexes. Then, 100 μL of the transfection mixture was added dropwise to each well of a 6-well plate, followed by gentle mixing. After 6 h of incubation, the transfection medium was replaced with complete culture medium, and cells were returned to standard culture conditions. Experimental groups were established as follows: *ODC* overexpression group (EGFP-ODC), *ODC* knockdown group 1 (si-523), *ODC* knockdown group 2 (si-910), EGFP empty vector control group (expressing EGFP only), and siRNA negative control group (NC). Total RNA was extracted 48 h post-transfection, and the relative expression of *ODC* was detected by RT-qPCR to select the siRNA with optimal interference efficiency.

### 2.7 Subcellular localization analysis

GCs were seeded in 35 mm confocal dishes. Upon reaching 80% confluence, the EGFP-ODC vector was transfected into the cells. After 48 h of culture, the medium was discarded, and cells were incubated with Mito Tracker Red CMXRos working solution at 37 °C for 15 min to stain mitochondria. Cells were washed with PBS, air-dried, fixed with 4% paraformaldehyde for 30 min, and washed three times. Cells were then permeabilized with PBS containing 0.3% Triton X-100 at room temperature for 20 min and washed twice. After blocking with 5% BSA at room temperature for 30 min and washing twice, nuclei were stained with DAPI for 15 min. Finally, cells were washed with PBS and imaged using a laser scanning confocal microscope (FV1000, Olympus, Tokyo, Japan).

### 2.8 Cell counting Kit-8 assay

GCs were seeded into 96-well plates and cultured until reaching 80% confluence, followed by transfection with EGFP-ODC, EGFP, si-910, or NC, respectively. Cell proliferation was assessed at 0, 24, 48, and 72 h post-transfection using the Cell Counting Kit-8 (CCK-8) assay (Beyotime Biotechnology, Shanghai, China). Briefly, 10 μL of CCK-8 solution was added to each well, followed by incubation in a 37 °C humidified incubator in the dark for 1 h. Absorbance at 450 nm was then measured using a microplate reader.

### 2.9 EdU cell proliferation assay kit

GCs were seeded into 35-mm confocal dishes and cultured until reaching 80% confluence, followed by transfection with EGFP-ODC, EGFP, si-910, or NC, respectively. Cell proliferation was assessed 48 h post-transfection using the EdU Proliferation Kit (Beyotime Biotechnology, Shanghai, China). EdU working solution was added to cultured GCs at room temperature, and the cells were incubated in the dark for 2 h. After discarding the medium, cells were fixed with 4% paraformaldehyde for 15 min at room temperature and washed three times. Cells were permeabilized with PBS containing 0.3% Triton X-100 for 15 min and rinsed twice. Click reaction solution was added, and cells were incubated for 30 min in the dark at room temperature. Nuclei were stained with DAPI, and cells were observed under a confocal microscope (FV1000, Olympus, Tokyo, Japan). EdU-positive cells were quantified using ImageJ software (V1.52a, NIH, Bethesda, MD, USA).

### 2.10 Cell cycle analysis

GCs were seeded in 100 mm dishes. When cell growth reached approximately 70%, cells were transfected with the overexpression vector EGFP-ODC or interference RNA si-ODC. Cell cycle distribution was analyzed using a cell cycle analysis kit (Beyotime) according to the manufacturer’s instructions. Cells were harvested 48 h post-transfection and washed three times with cold PBS. After fixation in 70% ice-cold ethanol, cells were stained with a mixture of Propidium Iodide (PI) and RNase A for 30 min at 37 °C in the dark. Data were acquired on a BD FACSCelesta flow cytometer (BD Biosciences, San Diego, CA, USA) and analyzed using FlowJo software (v10.0.7, Tree Star, Ashland, OR, USA).

### 2.11 ELISA for steroid hormones

GCs were seeded in 35-mm confocal dishes to 80% confluence and transfected with EGFP-ODC, EGFP, si-910, or NC. At 48 h post-transfection, supernatants were collected and P4/E2 levels were measured using ELISA kits (Quanzhou Jiubang Biotechnology) per manufacturer’s instructions. Biotinylated antigen was added to all wells except blanks, followed by incubation at 37 °C for 60 min. After three washes, enzyme-conjugated avidin was added and incubated at 37 °C for 30 min. Following washing, chromogenic substrate A and B were added and incubated at 37 °C in the dark for 15 min before termination with stop solution. OD was measured at 450 nm using a Model 680 microplate reader (Bio-Rad, Hercules, CA, USA).

### 2.12 Data analysis

Statistical analyses were performed using GraphPad Prism 5 software (GraphPad Software Inc., La Jolla, CA, USA). Statistical significance between two groups was determined using a two-tailed unpaired t-test, while multi-group comparisons were analyzed using One-Way ANOVA. All data were verified for normal distribution using the Shapiro–Wilk test; upon passing the homogeneity of variance test, appropriate statistical methods were applied. A *p*-value < 0.05 was considered statistically significant. Data are presented as mean ± standard error of the mean (SEM). Tissue expression analysis experiments included six biological replicates, and cell culture experiments included three biological replicates.

## 3 Results

### 3.1 Molecular identification and characterization of ODC

**Figure 1:**
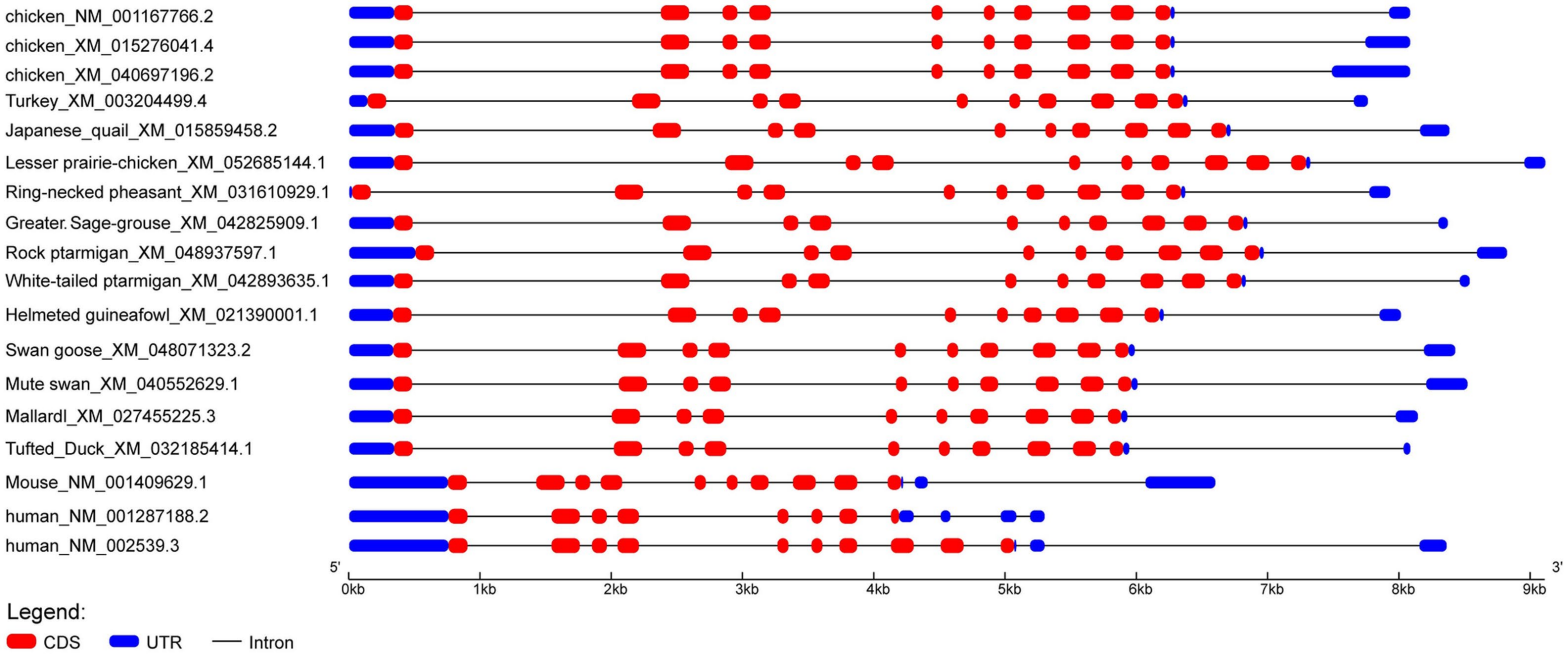
Transcriptional region structure of the *ODC* gene in Phasianidae and non-Phasianidae species.

We cloned the *ODC* gene transcript from the ovarian tissue of 300-day-old laying Wuding chickens (Supplementary Figure S1). The genomic sequence of the Wuding chicken *ODC* gene comprises 11 exons and 10 introns, with a full-length CDS of 1,395 bp encoding a polypeptide of 464 amino acid residues (Supplementary Figure S2). To investigate the transcriptional region structure of the Wuding chicken *ODC* gene, *ODC* transcript sequences from Phasianidae and non-Phasianidae species were downloaded from the NCBI database and visually analyzed. The study revealed that among all Phasianidae species, all species possess multiple transcript variants, with the exception of the turkey, which has only one. Notably, different transcript variants within the same species share an identical CDS sequence (Figure 1 and Supplementary Table S4). This indicates that the catalytic activity of ODC among these transcript variants remains consistent with its core enzymatic functions, with differences primarily located in the 5′ untranslated region (UTR). These findings suggest their potential involvement in post-transcriptional regulation of *ODC* expression, rather than altering the protein’s intrinsic functions.

**Figure 2:**
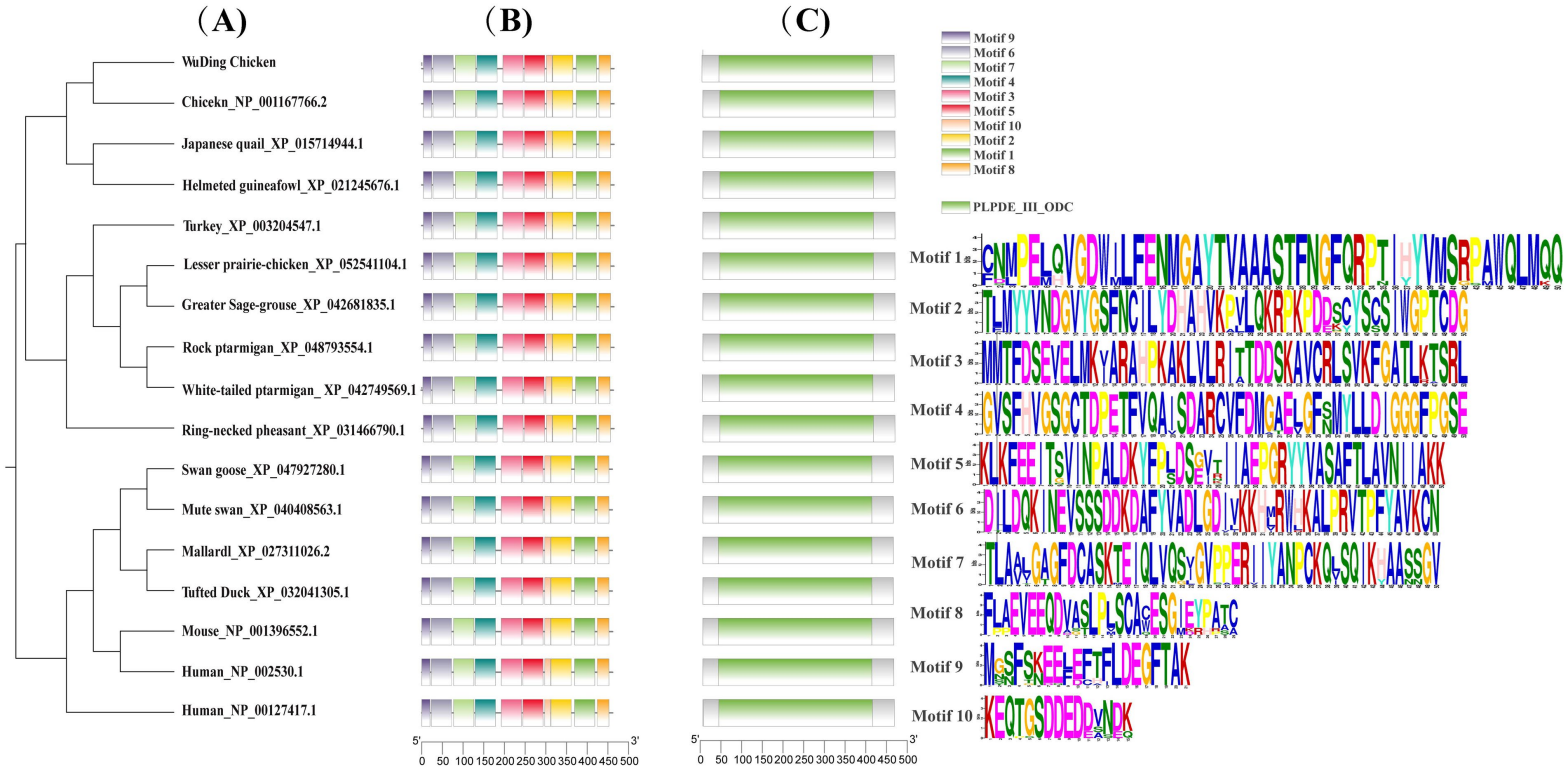
Phylogenetic tree, motif composition, and conserved domains of ODC in Wuding chickens and other species: **(A)** Phylogenetic tree; **(B)** motif composition; **(C)** conserved domains. The conserved motifs and domains in ODC are marked with differently colored boxes.

**Figure 3:**
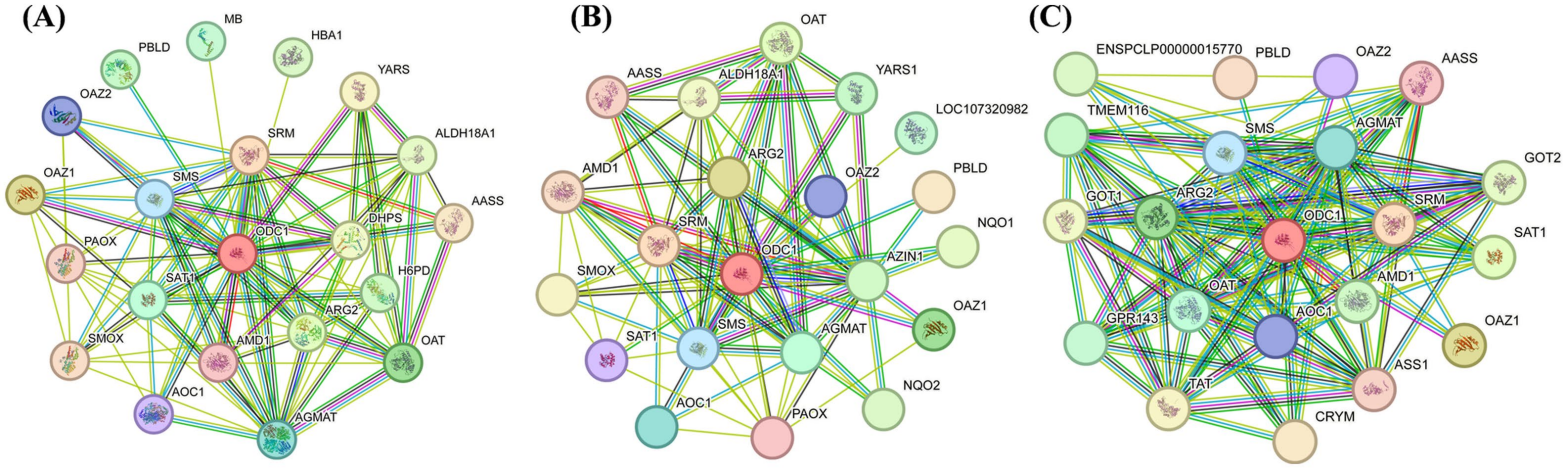
Protein-protein interaction network of ODC in Phasianidae species. **(A)** Chicken; **(B)** Japanese quail; **(C)** Ring-necked pheasant. Blue lines indicate interactions from curated databases. Pink lines indicate experimentally determined interactions. Green lines indicate gene neighborhood. Red lines indicate gene fusions. Dark blue lines indicate gene co-occurrence.

Multiple sequence alignment analysis showed that the Wuding chicken ODC amino acid sequence shares 100 and 99.86% homology with chicken *ODC* sequences in the NCBI database (XM_046914165.1 and NM_001167766.2, respectively), and 95.2 to 97.13% homology with *ODC* sequences of other Phasianidae species (Supplementary Figure S3). Phylogenetic analysis indicated that Wuding chicken *ODC* shares over 98% homology with Phasianidae species such as *Gallus gallus* (Red Junglefowl), Japanese quail, and turkey, clustering within the same clade (Figure 2). Physicochemical property analysis revealed that the Wuding chicken ODC protein is a hydrophilic protein containing four potential post-translational modification sites (Supplementary Table S5), lacking a signal peptide or transmembrane structure. Analysis of secondary and tertiary structures, domains, and motifs showed that the Wuding chicken ODC protein shares similar secondary and tertiary structures with other Phasianidae species and contains 10 motifs and one conserved PLPDE_III_ODC domain (Supplementary Tables S6, S7; Figure 2 and Supplementary Figure S4). The conserved PLPDE_III_ODC domain belongs to the type III PLP-dependent enzyme family; its activation catalyzes ornithine decarboxylation, making it a critical structure of ODC. Protein–protein interaction analysis of ODC proteins from three different Phasianidae species revealed interactions primarily with key proteins in the polyamine synthesis and catabolism pathways, such as spermine synthase (SMS), spermidine synthase (SRM), and polyamine oxidase (PAOX) (Figure 3).

### 3.2 Tissue expression analysis of ODC

**Figure 4:**
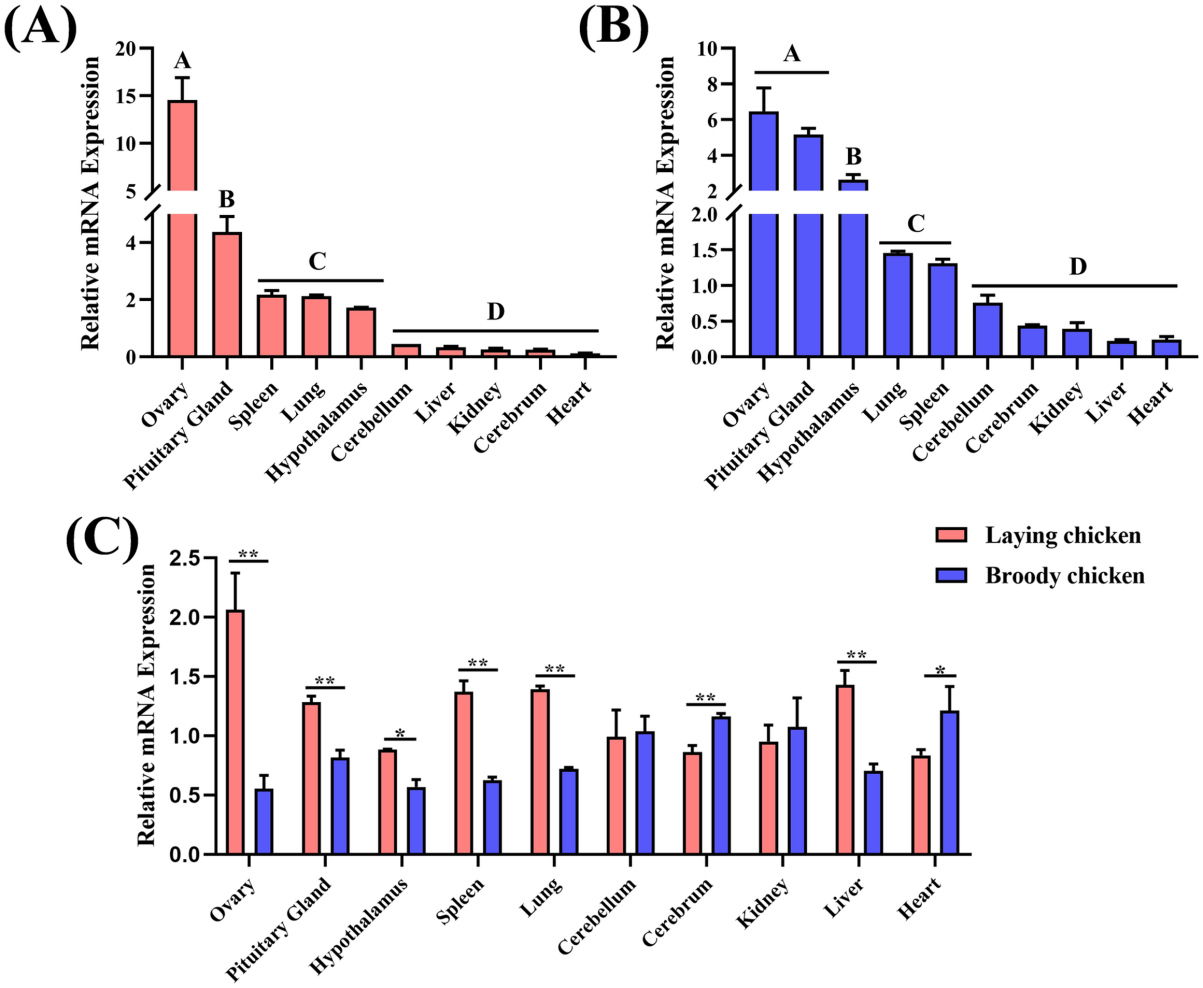
The mRNA expression profiles of *ODC* in Wuding chickens. **(A)** mRNA expression of *ODC* in various tissues of laying hens. **(B)** mRNA expression of *ODC* in various tissues of broody hens. **(C)** Expression differences of *ODC* in the same tissue at different stages. Statistical significance was assessed using a two-tailed independent-samples *t*-test for pairwise comparisons **(C)**, and one-way ANOVA followed by Tukey’s post-hoc test for multiple comparisons **(A,B)**. Results are presented as mean ± SEM from *n* = 6 independent individuals. *p < 0.05, **p < 0.01. Different letters **(A–D)** indicate significant differences (*p* < 0.05) in mRNA expression.

**Figure 5:**
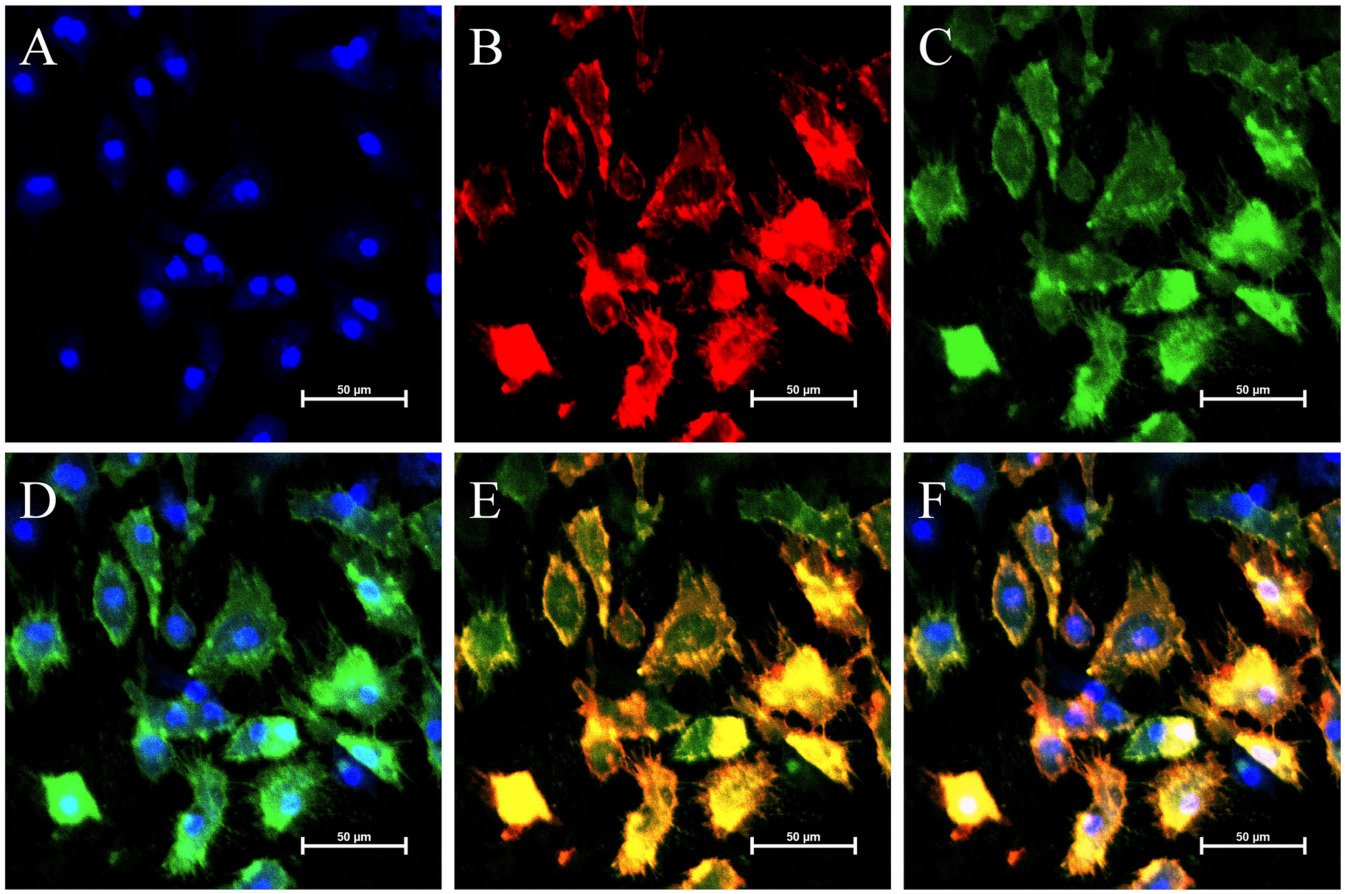
Subcellular localization of ODC in GCs, with the nucleus (blue), mitochondria (red), and EGFP-ODC (green) observed via LSCM. The nucleus **(A)** and mitochondria **(B)** were stained with DAPI and MitoTracker, respectively. **(C)** Green fluorescence represents EGFP-ODC expression. **(D)** Merged image of the nucleus and EGFP-ODC. **(E)** Merged image of mitochondria and EGFP-ODC. **(F)** Merged image showing colocalization of the nucleus, mitochondria, and EGFP-ODC. Scale bar = 50 μm.

In this experiment, the expression of the Wuding chicken *ODC* gene in different tissues during broody and laying periods was determined via RT-qPCR. The results demonstrated that the ODC gene was expressed in all ten tissues tested under both physiological states, although expression levels varied. During the laying period, *ODC* expression was highest in the ovary, followed by the pituitary, spleen, lung, and hypothalamus, with lower levels in other tissues (Figure 4A). In the broody period, *ODC* expression was highest in the ovary and pituitary, followed by the hypothalamus, lung, and spleen in decreasing order (Figure 4B). These expression patterns confirm the pivotal role of ODC in ovarian physiological processes. Furthermore, *ODC* expression in the ovary (*p* < 0.01), pituitary (*p* < 0.01) and hypothalamus (*p* < 0.05) was significantly higher during the laying period compared to the broody period (Figure 4C). These results demonstrate that ODC not only functions locally in the ovary but also participates in the neuroendocrine regulation of the HPG axis (see Figure 5).

### 3.3 Subcellular localization of ODC

To visually observe the cytoplasmic localization of ODC, Mito-Tracker Red CMXRos was used to label mitochondria as an indicator of the cytoplasm. We observed that the EGFP-ODC fusion protein (green) showed no co-localization with the nucleus (DAPI, blue), but exhibited diffuse cytoplasmic distribution (Figure 5). This indicates that ODC is localized in the cytoplasm, consistent with its function as an intracellular metabolic enzyme, and aligns with bioinformatics predictions and findings in other species.

### 3.4 ODC promotes GC proliferation via the PI3K/Akt/mTOR Signaling pathway

**Figure 6:**
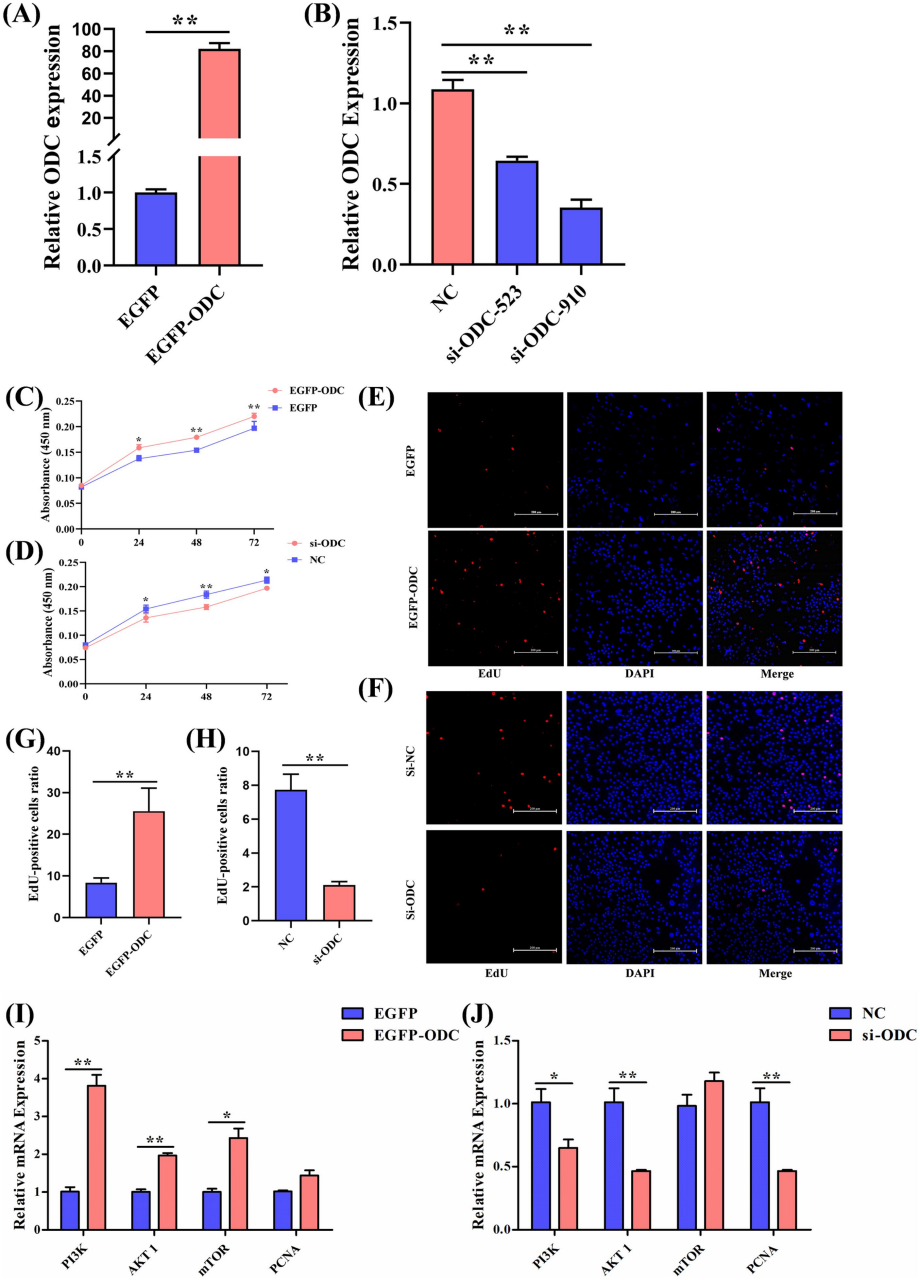
ODC promotes the proliferation of chicken GCs. **(A,B)** Relative mRNA expression levels of *ODC* in GCs 48 h after transfection with the *ODC* overexpression plasmid (EGFP-ODC) or si-ODC plasmids. **(C,D)** Proliferation rates of GCs assessed by CCK-8 assay following *ODC* overexpression and knockdown. **(E,F)** EdU fluorescence microscopy images of proliferating GCs after *ODC* overexpression and knockdown. **(G,H)** EdU assay analysis of proliferating GCs after *ODC* overexpression and knockdown. **(I,J)** mRNA expression levels of proliferation-related genes in GCs transfected with *ODC* overexpression or knockdown constructs. Two-tailed unpaired Student’s *t*-test was used for significance analysis. Data are presented as mean ± SEM (*n* = 3). *p < 0.05, **p < 0.01.

After transfecting the constructed *ODC* overexpression vector (EGFP-ODC) into follicular GCs for 48 h, mRNA expression of *ODC* gene was significantly upregulated by approximately 80-fold (Figure 6A). This confirms the successful transfection of the *ODC* overexpression vector, making it suitable for subsequent overexpression experiments. Simultaneously, si-ODC-523 and si-ODC-910 were transfected into follicular GCs. As shown in Figures 6B, 48 h post-transfection, mRNA levels of *ODC* gene decreased by approximately 40% in the si-ODC-523 group and 60% in the si-ODC-910 group compared to the NC group. Since si-ODC-910 exhibited higher interference efficiency, it was selected as the interfering RNA for subsequent knockdown experiments.

Cell proliferation of follicular GCs was assessed using the CCK-8 kit at 0 h, 24 h, 48 h, and 72 h following *ODC* overexpression and knockdown. Results showed that *ODC* overexpression significantly promoted GC proliferation at 24, 48, and 72 h post-transfection, whereas *ODC* knockdown significantly inhibited it (Figures 6C,D). Consistent with CCK-8 results, EdU assays showed an increased number of proliferating GCs after *ODC* overexpression, while knockdown significantly reduced proliferation (*p* < 0.01, Figures 6E–H). To further elucidate the molecular mechanism by which ODC promotes cell proliferation, we examined the expression levels of the cell proliferation marker *PCNA* and key genes of the classical growth regulatory PI3K/Akt/mTOR signaling pathway (*PI3K*, *AKT1*, *mTOR*) following *ODC* overexpression and knockdown. The results revealed that *ODC* overexpression significantly upregulated the expression of *PI3K* (*p* < 0.01), *AKT1* (*p* < 0.01), and *mTOR* (*p* < 0.05). Conversely, *ODC* knockdown significantly inhibited the expression of *PI3K* (*p* < 0.05), *AKT1* (*p* < 0.05), and *PCNA* (*p* < 0.01) (Figures 6I,J). These findings indicate that the ODC promotes GC proliferation.

### 3.5 ODC accelerates G1/S phase transition by regulating cyclin expression

**Figure 7:**
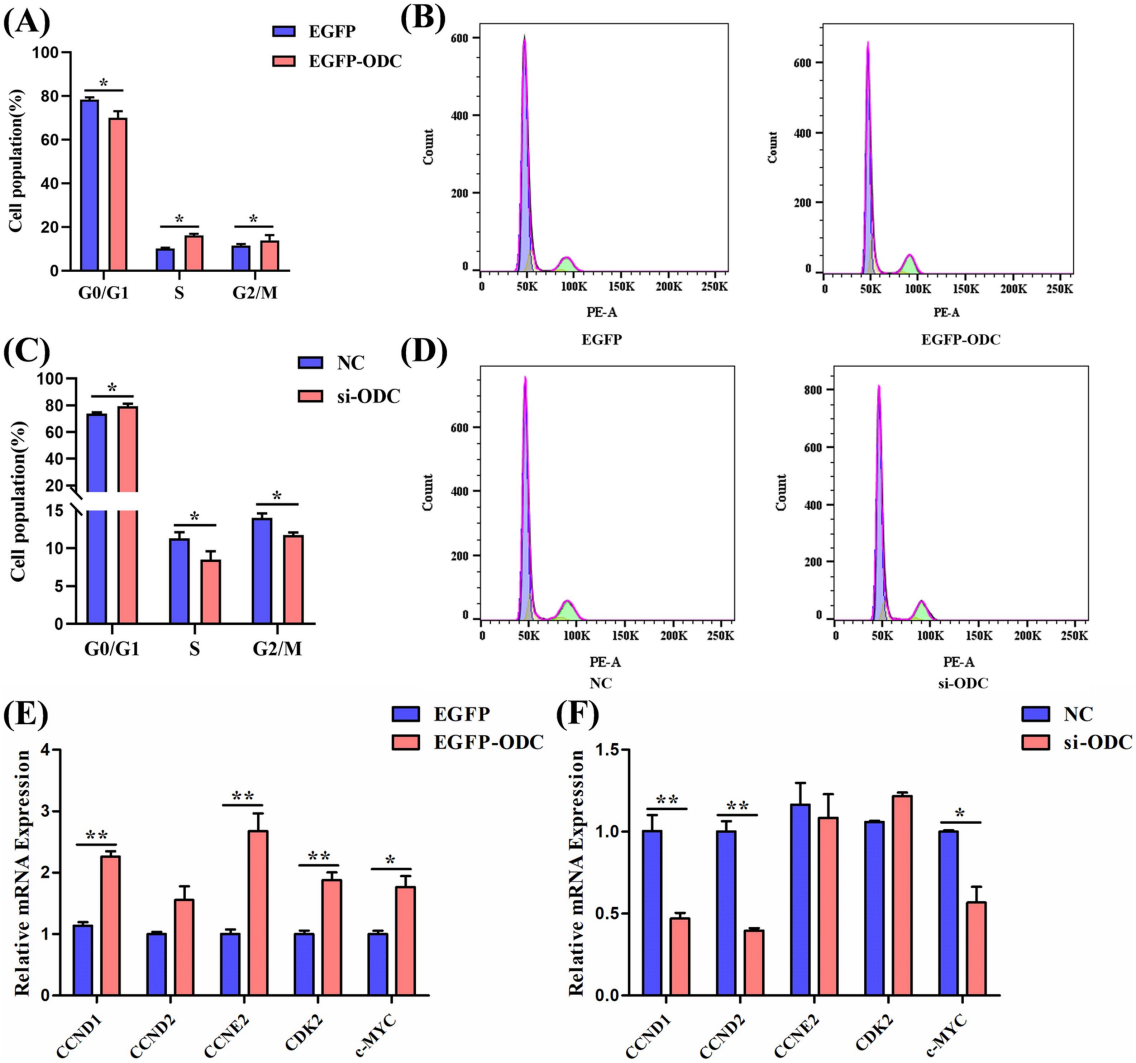
ODC promotes cell cycle progression in chicken GCs. **(A,B)** Flow cytometry analysis of cell cycle distribution in GCs after *ODC* overexpression. **(C,D)** Flow cytometry analysis of cell cycle distribution in GCs following *ODC* knockdown. **(E,F)** mRNA expression levels of cell cycle-related genes after overexpression and knockdown of *ODC* in transfected GCs. **(E,F)** Representative flow cytometry histograms showing DNA content (PE-A) on the x-axis and cell counts on the y-axis. The first peak from the left (purple) represents cells in the G0/G1 phase (2n DNA content), the intermediate region (yellow) represents the S phase, and the second peak (green) represents the G2/M phase (4n DNA content). Two-tailed unpaired Student’s t-test was used for significance analysis. Data are presented as mean ± SEM (*n* = 3). *p < 0.05, **p < 0.01.

To investigate whether ODC promotes proliferation via cell cycle regulation, flow cytometry was used to analyze cell cycle distribution. The flow cytometric histograms (Figures 7B,D) intuitively illustrate the DNA content distribution across cell cycle phases. Specifically, *ODC* overexpression facilitated the transition from G0/G1 to S and G2/M phases, evidenced by a diminished G0/G1 peak and a concomitant elevation in the S/G2/M populations. Conversely, siRNA-mediated knockdown resulted in a stark accumulation of cells in the G0/G1 peak, indicating a cell cycle arrest that underpins the observed suppression of granulosa cell proliferation (Figures 7A–D). To determine whether ODC regulates cell cycle progression via modulation of cell cycle-related gene expression in GCs, we quantified mRNA expression levels of key cell cycle genes by RT-qPCR. The results demonstrated that *ODC* overexpression significantly upregulated the expression levels of cell cycle-related genes *c-MYC* (*p* < 0.05), *CCND1* (*p* < 0.01), *CCND2* (*p* < 0.05), *CCNE2* (*p* < 0.01), and *CDK2* (*p* < 0.01) (Figure 7E). In contrast, *ODC* knockdown significantly downregulated the expression of *c-MYC* (*p* < 0.05), *CCND1* (*p* < 0.01), and *CCND2* (*p* < 0.01) (Figure 7F). The concurrent increase in S-phase cells alongside upregulation of key cell cycle regulators (Figure 7F) demonstrates that ODC facilitates the G0/G1-to-S phase transition, thereby enhancing granulosa cell proliferative capacity.

### 3.6 ODC inhibits GC apoptosis

**Figure 8:**
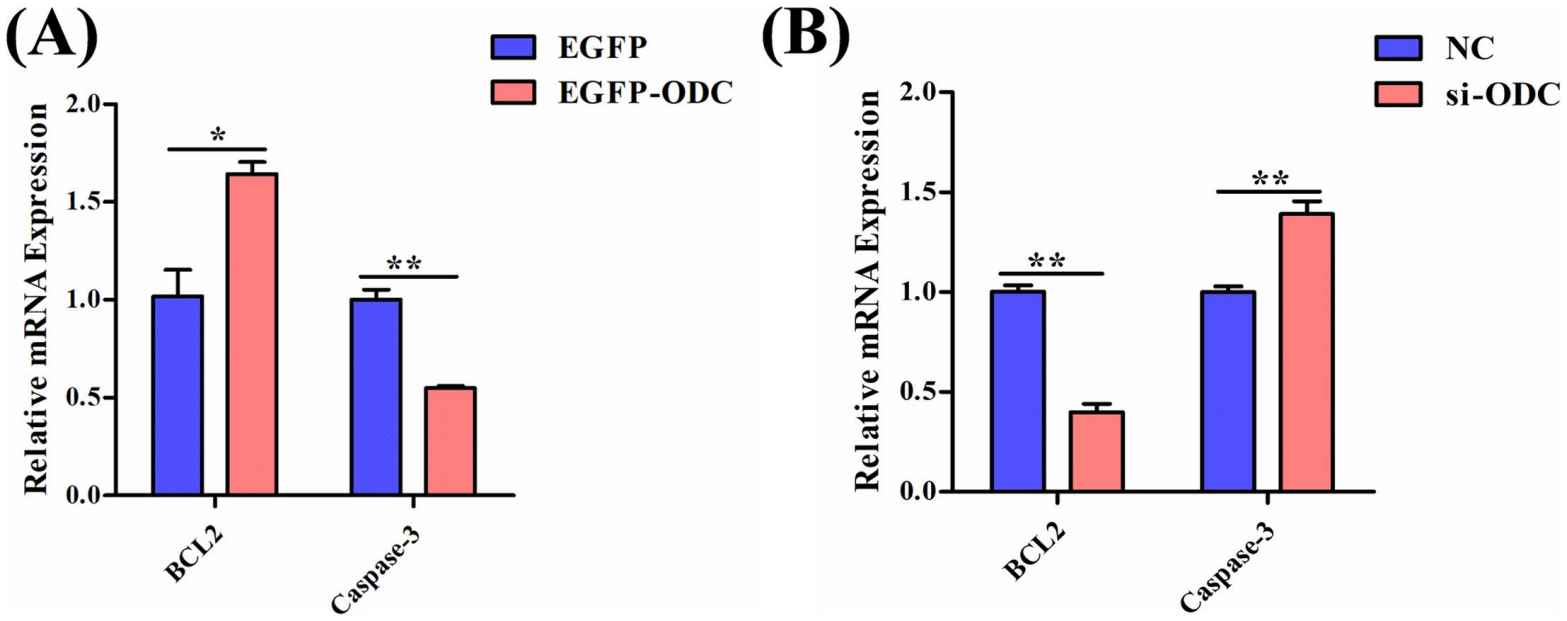
ODC inhibits apoptosis in chicken GCs. The mRNA expression levels of apoptosis-related genes in GCs transfected with *ODC* overexpression **(A)** or knockdown constructs **(B)**. Two-tailed unpaired Student’s *t*-test was used for significance analysis. Data are presented as mean ± SEM (*n* = 3). *p < 0.05, **p < 0.01.

Follicular atresia is primarily caused by GC apoptosis. To further explore the role of ODC in follicular atresia, we investigated the relationship between the *ODC* gene and apoptosis by examining the mRNA expression levels of apoptosis-related genes. Results showed that *ODC* overexpression significantly upregulated the mRNA expression of the anti-apoptotic gene *BCL2* (*p* < 0.05) while significantly downregulating the mRNA expression of the pro-apoptotic gene *Caspase-3* (*p* < 0.01) (Figure 8A). Conversely, *ODC* knockdown significantly inhibited *BCL2* expression (*p* < 0.01) and significantly increased *Caspase-3* mRNA levels (*p* < 0.01) (Figure 8B). These results indicate that ODC inhibits apoptosis in chicken ovarian GCs, thereby reducing follicular atresia.

### 3.7 ODC enhances steroid hormone synthesis capacity

**Figure 9:**
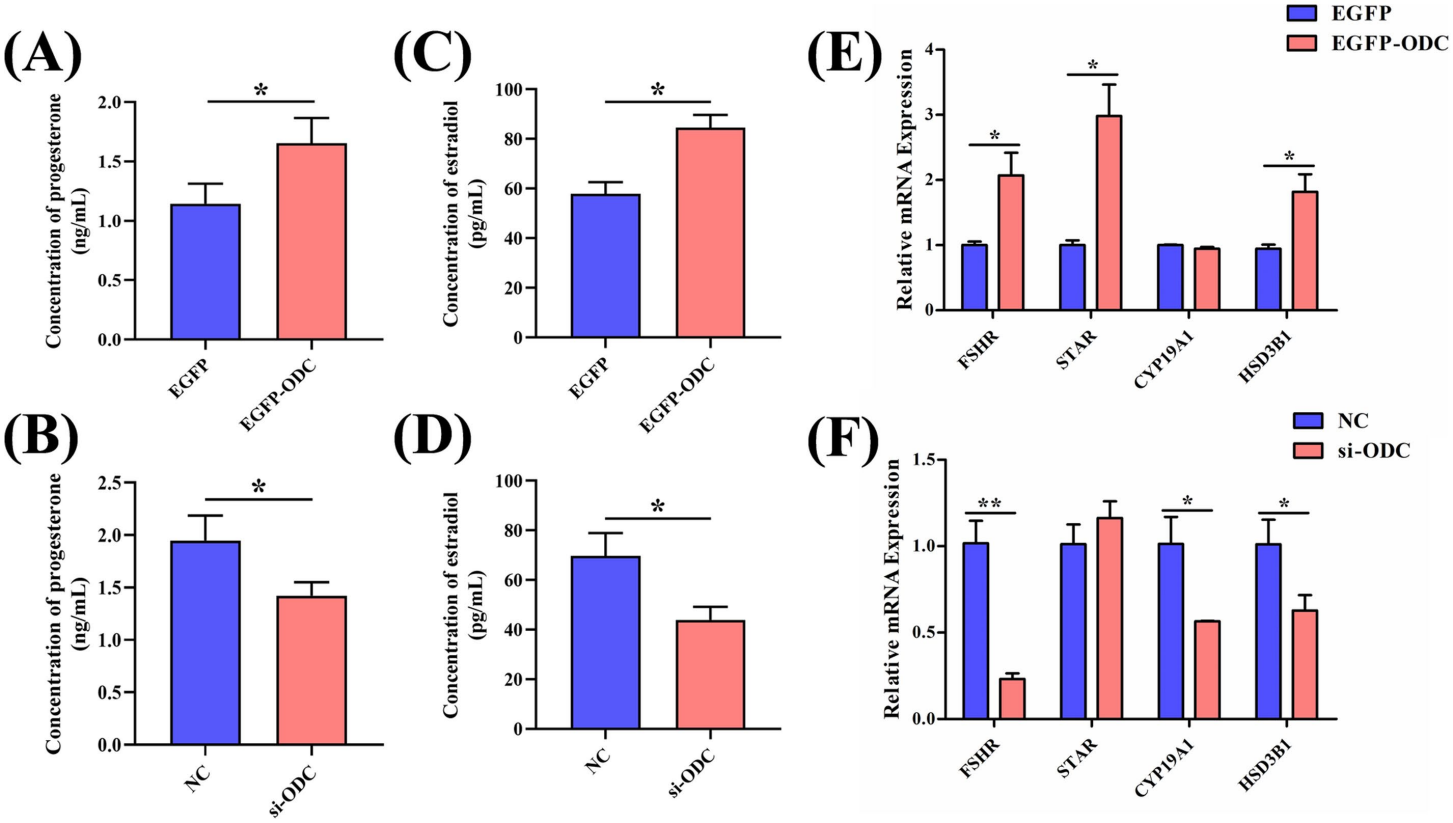
ODC facilitates the synthesis and secretion of steroid hormones in chicken GCs. **(A,B)** Effects of *ODC* overexpression and knockdown on P4 content in transfected GCs. **(C,D)** Effects of *ODC* overexpression and knockdown on E2 content in transfected GCs. **(E,F)** Effects of *ODC* overexpression and knockdown on the mRNA expression of steroidogenesis-related genes in transfected GCs. Two-tailed unpaired Student’s t-test was used for significance analysis. Data are presented as mean ± SEM (*n* = 3). *p < 0.05, **p < 0.01.

Sex hormones (E2 and P4) secreted by GCs are key regulators of follicle maturation and ovulation, and their synthesis efficiency directly determines the outcome of follicular development. To investigate the role of the *ODC* gene in sex hormone secretion during chicken follicular development, we performed overexpression and knockdown experiments using EGFP-ODC and si-ODC in primary cultured Wuding chicken GCs and assessed the effects on steroid hormone synthesis. ELISA assays demonstrated that overexpression of *ODC* significantly augmented the secretion levels of P4 and E2 (*p* < 0.05) (Figures 9A–D), whereas *ODC* knockdown markedly reduced their secretion levels (*p* < 0.05). Expression analysis of steroidogenesis-related genes revealed that *ODC* overexpression promoted the mRNA expression of *FSHR* (*p* < 0.05), *STAR* (*p* < 0.05), and *HSD3B1* (*p* < 0.01) in GCs (Figure 9E). However, *ODC* knockdown resulted in a significant reduction in the mRNA expression of *FSHR* (*p* < 0.01), *CYP19A1* (*p* < 0.05), and *HSD3B1* (*p* < 0.05) (Figure 9F). These findings demonstrate that ODC robustly enhances P4 and E2 biosynthesis and secretion by upregulating transcription of rate-limiting enzymes in the steroidogenic pathway. This mechanism mechanistically underpins hierarchical follicle selection in avian ovaries.

### 3.8 Feedback regulation of polyamine metabolism in GCs

**Figure 10:**
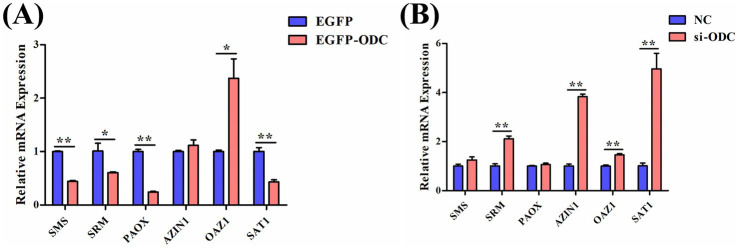
Regulatory effects of ODC on the polyamine metabolic flux. The mRNA expression levels of polyamine metabolism-related genes in GCs transfected with *ODC* overexpression **(A)** or knockdown constructs **(B)**. Two-tailed unpaired Student’s *t*-test was used for significance analysis. Data are presented as mean ± SEM (*n* = 3). *p < 0.05, **p < 0.01.

To explore whether ODC affects GC proliferation and apoptosis by altering intracellular polyamines, we investigated the effects of *ODC* overexpression and interference on the polyamine metabolic network in chicken GCs. The results revealed that *ODC* overexpression led to a significant decrease in the expression of *SRM* (*p* < 0.05), *SMS* (*p* < 0.01), *PAOX* (*p* < 0.01), and *SAT1* (*p* < 0.01), while the mRNA expression of *OAZ1* significantly increased (*p* < 0.05) (Figure 10A). Conversely, *ODC* interference resulted in significantly elevated mRNA expression of *SRM* (*p* < 0.01), *AZIN1* (*p* < 0.01), *OAZ1* (*p* < 0.05), and *SAT1* (*p* < 0.05) (Figure 10B). These findings establish that ODC regulates polyamine biosynthesis gene expression, leading to enhanced intracellular polyamine accumulation.

## 4 Discussion

This study provides the first systematic characterization of *ODC* gene expression dynamics across the HPG axis during the transition from the laying to brooding phase in Wuding chickens. Our findings reveal ODC as a key regulator, not merely a metabolic enzyme, governing reproductive plasticity in indigenous poultry breeds. This study revealed that *ODC* expression in the ovary and pituitary of Wuding chickens was significantly upregulated during the laying period, whereas a coordinated downregulation occurred in both tissues during the broody phase. These findings indicate that ODC regulates follicular development during the laying-to-broody transition through dual mechanisms: the neuroendocrine axis and ovarian autocrine/paracrine signaling networks. In the ovary, elevated *ODC* expression directly drives GC proliferation and stimulates steroid hormone secretion, thereby sustaining follicular growth. Meanwhile, in the pituitary (the core endocrine component of the hypothalamic–pituitary-gonadal axis), upregulated *ODC* enhances gonadotrope function, maintains FSH/LH synthesis and secretion, and provides sustained gonadotropic stimulation to ovarian GCs, thereby amplifying their proliferative capacity (16). Conversely, the coordinated *ODC* downregulation in the pituitary and ovary during the broody phase may synergistically suppress GC proliferation through both indirect neuroendocrine inhibition (reduced FSH/LH secretion) and direct ovarian suppression (impeded GC proliferation), ultimately triggering follicular atresia and ovarian regression in broody Wuding chickens. This aligns with mammalian models where reduced ODC activity impedes embryonic development (17), highlighting the evolutionary conservation of polyamine-dependent reproductive functions.

GCs, as the core functional unit of follicular architecture, regulate follicular development and ovulatory cycles through the synthesis of steroid hormones such as E2 and P4 (18), with their proliferative capacity directly determining follicle selection and maturation. Proliferating cell nuclear antigen (PCNA), a widely recognized biomarker of cell proliferation, plays essential roles in DNA synthesis, damage repair, and cell cycle progression (19). The PI3K/Akt/mTOR signaling pathway, a central regulator of cell growth and metabolism, drives protein synthesis and proliferation through phosphorylation of p70S6K and 4E-BP1 (20). In this study, altered expression of *PCNA* and key genes in the PI3K/Akt/mTOR pathway indicated that ODC promotes GC proliferation, a conclusion further supported by EdU and CCK-8 assay results. Among apoptosis-related genes, BCL2 exerts anti-apoptotic effects, whereas Caspase-3 acts as a critical executor of apoptosis. Previous studies have demonstrated that FSH induction increases *BCL2* expression and reduces *Caspase-3* levels, thereby suppressing GC apoptosis (21). Herein, ODC inhibited GC apoptosis by upregulating *BCL2* and downregulating *Caspase-3*. Integrating the differential expression of ODC between the laying and broody periods, we hypothesize that during the laying phase, ODC sustains GC proliferation and suppresses apoptosis to meet the demands of rapid follicular expansion. Conversely, the reduced expression of *ODC* during the broody period restricts GC proliferation, thereby promoting follicular atresia.

A pivotal finding of this study is the identified feedback between ODC and c-MYC. Traditionally, c-MYC is viewed as a transcriptional activator of ODC (22). However, our results indicate that ODC also sustains *c-MYC* levels, likely through a translational mechanism. We propose that ODC-driven spermidine synthesis is required for eIF5A hypusination, a critical post-translational modification (23). Hypusinated eIF5A is indispensable for translating polyproline motif-containing proteins, which exhibit ribosomal stalling during synthesis. c-MYC contains a polyproline-rich domain, and its translation requires functional eIF5A (24). Previous studies have demonstrated that hypusination of eIF5A enhances the translational level of MYC in human colorectal cancer cells (25). Thus, the upregulation of *c-MYC* in *ODC*-overexpressing GCs may be a downstream consequence of elevated spermidine levels enhancing *eIF5A* translational efficiency. The synthesized c-MYC relocalizes to the nucleus, where it transcriptionally activates target genes such as Cyclin D1 and Cyclin E2 (26). This correlates with accelerated G1/S phase transition, thereby promoting GC proliferation to sustain follicular hierarchy development. Conversely, in the *ODC*-knockdown group, the depletion of polyamines may impair eIF5A hypusination, resulting in ribosomal stalling during *c-MYC* mRNA translation. The observed reduction in c-MYC protein and downstream downregulation of *Cyclin/CDK* complexes correlate with G0/G1 cell cycle arrest, suggesting a molecular association with follicular stasis during the broody phase.

The transition from a productive laying state to broodiness in Wuding chickens is characterized by profound ovarian remodeling. Our data demonstrate that GCs in hierarchical follicles adopt a “pro-proliferative metabolic adaptation” orchestrated by ODC. Spermidine/spermine N1-acetyltransferase (SAT1) and acetylated polyamine oxidase (PAOX) are key enzymes in the polyamine acetylation and degradation pathway, which catalyze the conversion of spermidine and spermine into putrescine and facilitate the extracellular export of excess putrescine (27). While traditional homeostatic models suggest that high polyamine levels should trigger catabolic enzymes like SAT1 and PAOX to prevent toxicity, we observed a paradoxical downregulation of these genes upon *ODC* overexpression. This shift suggests that rapidly proliferating GCs prioritize the sequestration of polyamines over their degradation—a phenomenon analogous to the Warburg effect, where biosynthetic requirements outweigh catabolic homeostasis (28). Polyamines stabilize DNA structure and mediate DNA repair during cell proliferation, thereby protecting the genome from genotoxic stress (29). Furthermore, the action of SAT1 and PAOX generates hydrogen peroxide (H2O2) and reactive aldehydes (acrolein) as byproducts (21). The accumulation of these reactive oxygen species (ROS) is a known trigger for ferroptosis and apoptosis. To meet this demand, these cells appear to actively suppress the catabolic pathway (SAT1/PAOX) to prevent the degradation of *de novo* synthesized polyamines. These findings indicate that elevated ODC activity during the laying phase maintains polyamine homeostasis by suppressing *SAT1* and *PAOX* expression. This mechanism may promote DNA stability during GC proliferation and attenuate ROS-mediated apoptotic damage by reducing hydrogen peroxide (H_2_O_2_) and acrolein accumulation. This suppression may be directly mediated by c-MYC, which is known to repress *SAT1* transcription in hyper-proliferative states (30).

Moreover, this study identifies ODC as an essential regulator for GCs to achieve maximal steroidogenic capacity. *ODC* overexpression upregulated *STAR*, *Hydroxy-Δ^5^-steroid dehydrogenase 3 beta-1 (HSD3B1)* and *Cytochrome P450 aromatase (CYP19A1)* expression, whereas knockdown downregulated these genes, consistent with NTRK2-mediated steroidogenic enhancement (31). ODC activity may facilitate spermidine biosynthesis and eIF5A hypusination, a post-translational modification that specifically enhances the translational efficiency of steroidogenic genes, thereby elevating *STAR*, *HSD3B1*, and *CYP19A1* expression (14). Concurrently, *ODC* overexpression upregulated *Follicle-stimulating hormone receptor (FSHR)* expression, increasing GC sensitivity to FSH and amplifying cAMP/PKA signaling to potentiate steroidogenic regulation (32). This regulatory pattern may involve dual processes: elevated FSHR potentiates FSH signal responsiveness, while increased STAR accelerates cholesterol translocation to mitochondria, collectively driving E2 and P4 biosynthesis (33). During the laying phase, elevated ODC activity sustains steroidogenic pathway integrity for dominant follicle development. Conversely, reduced *ODC* expression in the broody period disrupts this network, leading to steroidogenic insufficiency and initiating follicular atresia, thereby establishing ODC as a critical metabolic checkpoint in hormone-dependent folliculogenesis.

As an endogenous inhibitor of ODC, ornithine decarboxylase antizyme 1 (OAZ1) mediates proteasomal degradation of ODC by forming an AZ-ODC complex (34). However, we observed that *OAZ1* expression is independent of *ODC* levels and is instead modulated by alternative regulatory mechanisms. Notably, antizyme inhibitor 1 (AZIN1), which competitively binds OAZ1 with high affinity, antagonizes OAZ1-mediated ODC degradation (35). Intriguingly, *AZIN1* is upregulated exclusively upon *ODC* knockdown, revealing its role as a compensatory protector that preserves basal polyamine levels required for cell survival under conditions of ODC inhibition.

In conclusion, this study elucidates the pivotal regulatory role of ODC in avian follicular development. We demonstrate that ODC coordinates the laying-to-broody transition in Wuding chickens via the spermidine–eIF5A axis, which drives c-MYC/Cyclin D–mediated GC proliferation and modulates FSHR/STAR-dependent steroidogenesis, thereby determining follicular fate. Building upon these findings, future research will further extend these mechanisms to a physiological level by: (1) quantifying polyamine metabolic flux using HPLC/LC–MS to refine the link between polyamine homeostasis and steroidogenic capacity; (2) further validating the spermidine-eIF5A-c-MYC axis through GC7-mediated inhibition; and (3) leveraging adenovirus-mediated targeted delivery to confirm these regulatory circuits in vivo during reproductive transitions.

## 5 Conclusion

This study, using Wuding chickens as a model, demonstrates through tissue expression profiling and functional in vitro assays that ODC acts as a critical regulator of the laying-to-broody physiological transition in chickens. During the laying phase, elevated *ODC* expression in ovarian tissues sustains relatively high spermidine levels through pro-proliferative metabolic adaptation. This promotes eIF5A hypusination, which activates the c-MYC–Cyclin signaling axis, revealing a metabolic-proliferation correlation linked to GC proliferation and apoptosis suppression. Concurrently, ODC enhances steroidogenesis by transcriptionally upregulating *FSHR* and *STAR*. Conversely, during brooding, downregulation of *ODC* modulates the transcription of *SAT1* and *PAOX*, potentially triggering ROS accumulation (H_2_O_2_, acrolein), which accelerates GC apoptosis. These findings refine the avian HPG axis regulatory framework, elucidate the polyamine-mediated “metabolism-proliferation-secretion” feedback mechanism in GCs, and identify ODC as a actionable molecular target for improving egg production in indigenous chicken breeds through targeted molecular breeding strategies.

## Data Availability

The original contributions presented in the study are included in the article/Supplementary material, further inquiries can be directed to the corresponding authors.

## Glossary

ODC: Ornithine decarboxylase
GCs: Granulosa cells
LH: Luteinizing hormone
FSH: Follicle-stimulating hormone
E2: Estradiol
P4: Progesterone
GnRH: Gonadotropin-releasing hormone
STAR: Steroidogenic acute regulatory protein
eIF5A: eukaryotic translation initiation factor 5A
PCNA: Proliferating cell nuclear antigen
PI3K: Phosphatidylinositol 3-kinase
AKT1: AKT serine/threonine kinase 1
mTOR: Mammalian target of rapamycin
CCND1: Cyclin D1
CCND2: Cyclin D2
CCNE2: Cyclin E2
CDK2: Cyclin-dependent kinase 2
c-MYC: v-myc avian myelocytomatosis viral oncogene homolog
BCL2: B-cell lymphoma 2
Caspase-3: Cysteine-aspartic acid protease 3
FSHR: Follicle-stimulating hormone receptor
HSD3B1: 3β-Hydroxysteroid dehydrogenase
CYP19A1: Cytochrome P450 family 19 subfamily A member 1
SMS: spermine synthase
SRM: spermidine synthase
PAOX: polyamine oxidase
SAT1: spermidine/spermine N1-acetyltransferase 1
OAZ1: ornithine decarboxylase antizyme 1
AZIN1: antizyme inhibitor 1
cAMP: Cyclic adenosine monophosphate
PKA: Protein kinase A
GAPDH: Glyceraldehyde-3-phosphate dehydrogenase
HPG: Hypothalamic–pituitary-gonadal axis
RT-qPCR: Real-time fluorescence quantitative PCR
cDNA: Coding DNA sequence
siRNA: Small interfering RNA
EGFP: pEGFP-N1
CCK-8: Cell counting kit-8
EdU: 5-Ethynyl-2′-deoxyuridine
