# Supplementary material for: Ornithine decarboxylase influences granulosa cell proliferation and steroidogenesis: implications for ovarian regression in Wuding chickens
Source: Front Vet Sci. 2026 Apr 20;13:1801162. doi: 10.3389/fvets.2026.1801162 (PMC13139908; doi:10.3389/fvets.2026.1801162)
Supplement: Supplementary file 1 [file Table_1.docx]

**Supplementary Materials**

**Supplementary Table S1.** Information on primers used for the ODC gene in this study.

| Primer ID | | (5'→3')Primer sequence | Products length | GenBank ID | Annealing temperature/°C |
| --- | --- | --- | --- | --- | --- |
| ODC | | F:CTCAAGTCCTTCCACATAAACT  R:TGTCGTGTTTATGCCAAACCTA | 1567 | NM_001167766.2 | 53.5 |
| ODC-qPCR | | F:AAACCAGTTCTGCAAAAGCG  R:CATTAATTGCCATGCTGGTC | 243 | NM_001167766.2 | 53 |
| GAPDH-qPCR | | F:ATCTTCCAGGAGCGTGACC  R:CACGATGCATTGCTGACAA | 230 | NM_204305.2 | 53 |
| EGFP-ODC | | F:GAGCTCATGTCAGGAATCATGGGTAGC  R:GGTACCTACATTAATACTAGCTGAAGCAC | 1392 | NM_001167766.2 | 58.7 |
| SRM-qPCR | | F:CCTGTTGTGGAATACGCCTA | 206 | XM_015297174.3 | 60 |
|  |  | R:GCCTTCCGTGCAAACTCTGG |  |  |  |
| SMS-qPCR | | F:CAGAAAGTGACCTGGCATAT | 213 | NM_001030803.2 | 60 |
|  |  | R:CTAAGACATCTCCGCATGTT |  |  |  |
| PAOX-qPCR | | F:CCAGGTGGAGTGCGAGGATG | 253 | XM_015278694.4 | 60 |
|  |  | R:GGGCTCGGCAAGAGGTGATT |  |  |  |
| SAT1-qPCR | | F:ATCAAGTGGTGCTAACCGAG | 223 | NM_204186.2 | 60 |
|  |  | R:TCCAATGCCAAGTCCTCTGT |  |  |  |
| OAZ1-qPCR | | F:GTGCTCCTGATGTCCCTCACC | 186 | NM_204916.1 | 60 |
|  |  | R:TGTGTTTGGCATCTGTAAGC |  |  |  |
| AZIN1-qPCR | | F:AATACGGCTGAGATGAAAGG | 231 | NM_001008729.2 | 60 |
|  |  | R:AGTAGAATTGCATCTTACAG |  |  |  |
| CYP19A1-qPCR | | F:ATGTTCCATCACGCTATTT | 240 | NM_001001761.4 | 61.5 |
|  |  | R:GATTCTTGTTTGGGCTTCT |  |  |  |
| HSD3B1-qPCR | | F:GCCAAAGAGGAGCAAACCAGAG | 104 | NM_205118.2 | 60 |
|  |  | R:TCCAGCAGTAAGCGAACGATCC |  |  |  |
| STAR-qPCR | | F:TGCCATCTCCTACCAACA | 190 | NM_204686.3 | 61.5 |
|  |  | R:CATCTCCATCTCGCTGAAG |  |  |  |
| FSHR-qPCR | | F:GAGCGAGGTCTACATACAAG | 241 | NM_205079.2 | 61.5 |
|  |  | R:CGCATGCCATAATGGGAAAT |  |  |  |
| CCND1-qPCR | | F:CAGAAGTGCGAAGAGGAAGT | 187 | NM_001396513.1 | 60 |
|  |  | R:TGATGGAGTTGTCGGTGTAA |  |  |  |
| CCND2-qPCR | | F:CCCCAAGAGCTGCTGGAATG | 172 | NM_001397876.1 | 60 |
|  |  | R:TGGCACAAAGGGCAATGAAC |  |  |  |
| CCNE2-qPCR | | F:TGTGAATGTGGCAAGGAAAG | 164 | NM_001030945.2 | 61.5 |
|  |  | R:TTGACACGGGTGATAACTGA |  |  |  |
| CDK2-qPCR | | F:CCAGAACCTCCTCATCAAC | 171 | NM_001199857.2 | 61.5 |
|  |  | R:CAGATGTCCACAGCAGTC |  |  |  |
| PCNA-qPCR | | F:AGCACCAAATCAGGAAAAG | 177 | NM_204170.3 | 60 |
|  |  | R:GCACAGGAGATGACAACAG |  |  |  |
| MYC-qPCR | | F:GCCCACGACCAGCAGCGACT | 234 | NM_001030952.2 | 60 |
|  |  | R:TTGGCGGCTGGGTATTCCAC |  |  |  |
| PI3K-qPCR | F:CAGGTGGAGGCTATGGAGAAG | 196 | NM_001004410.2 | 57.5 |  |
|  | R:TGCACTCCTCAAGCCGAAG |  |  |  |  |
| AKT1-qPCR | | F:TGATGGCACATTCATTGGCTAC | 122 | NM_001396387.1 | 57.5 |
|  |  | R:TGTTTGGTTTAGGTCGTTCTGTCT |  |  |  |
| mTOR-qPCR | | F:GCACAAACCTCTGCGACAT | 250 | XM_040689168.2 | 62 |
|  |  | R:CGTGAGCAAGCGAGAACAA |  |  |  |
| BCL2-qPCR | | F:CAGAGGGACTTCGCCCAGAT | 235 | NM_205339.3 | 61.5 |
|  |  | R:TGTGCAGGTGCCGGTTCAG |  |  |  |
| Caspase3-qPCR | | F:AAAAGATGGACCACGCTCAG | 182 | NM_204725.2 | 60 |
|  |  | R:CTCGGTGGAAGTTCTTATTG |  |  |  |

**Supplementary Table S2.** Bioinformatics analysis tools used in this study.

| **Softwares** | **Online website** | **Analysis content** |
| --- | --- | --- |
| MegAlign |  | Sequence identity analysis |
| MEGA 12 |  | Phylogenetic tree construction |
| TBtools |  | Phylogenetic tree, motif, and conserved domain visualization analysis |
| NCBI | <https://www.ncbi.nlm.nih.gov/datasets/genome/> | Download of reference sequences and genome annotation files |
| ORF Finder | <https://www.ncbi.nlm.nih.gov/orffinder> | CDS identification |
| Gene Structure Display Server | <https://gsds.gao-lab.org/> | Structural visualization of transcribed regions |
| MEME Suite | <https://meme-suite.org/meme/> | Structural motif prediction |
| Conserved Domains | <https://www.ncbi.nlm.nih.gov/Structure/bwrpsb/bwrpsb.cgi> | Conserved domain prediction |
| PROSITE | <http://prosite.expasy.org/prosite.html> | Post-translational modification site prediction |
| ProtParam | <https://web.expasy.org/protparam/> | Physicochemical properties prediction |
| ProtScale | <https://web.expasy.org/protscale/> | Hydrophobicity prediction |
| SignalP 5.0 Server | <https://services.healthtech.dtu.dk/services/SignalP-5.0/> | Signal peptide prediction |
| TMHMM 2.0 Server | <https://services.healthtech.dtu.dk/services/TMHMM-2.0/> | Transmembrane domain prediction |
| PSORT | <https://www.genscript.com/psort.html> | Protein subcellular localization prediction |
| SOPMA | <http://npsa-pbil.ibcp.fr/> | Secondary structure prediction |
| SWISS-MODEL | <http://swissmodel.expasy.org/> | Tertiary structure prediction |
| STRING 12.0 | <https://cn.string-db.org/> | Protein-protein interaction prediction |
| InterPro | <https://www.ebi.ac.uk/interpro/> | Biological process and molecular function prediction |

**Supplementary Table S3.** Information on ODC interference primers (siRNAs).

| Primer ID | Primer sequence | Use of primers |
| --- | --- | --- |
| Si-ODC-523 | S:5’ GGAGCUACUCUCAAGACAA 3’ | ODC knockdown |
|  | A:5’ UUGUCUUGAGAGUAGCUCC 3’ |  |
| Si-ODC-910 | S:5’ GACAGGUUCUGAUGAUGAA 3’ | ODC knockdown |
|  | A:5’ UUCAUCAUCAGAACCUGUC 3’ |  |
| NC | S:5’ UUCUCCGAACGUGUCACGUTT 3’ | Negative control |
|  | A:5’ ACGUGACACGUUCGGAGAATT 3’ |  |

**Supplementary Table S4.** Structural information of ODC transcript regions in Phasianidae and non-Phasianidae species.

| **species** | **number of exon** | **number of intron** | **length/bp** | | | | | | | | | | | | | | |
| --- | --- | --- | --- | --- | --- | --- | --- | --- | --- | --- | --- | --- | --- | --- | --- | --- | --- |
|  |  |  | **CDS** | **5‘UTR** | **E1** | **E2** | **E3** | **E4** | **E5** | **E6** | **E7** | **E8** | **E9** | **E10** | **E11** | **E12** | **3'UTR** |
| **Chicken_NM_001167766.2** | **11** | **10** | **1395** | **192** | **160** |  | **146** | **174** | **173** | **135** | **82** | **84** | **163** | **113** | **215** | **484** | **342** |
| **Chicken_XM_015276041.4** | **11** | **10** | **1395** | **373** | **341** |  | **146** | **174** | **173** | **135** | **82** | **84** | **163** | **113** | **215** | **484** | **342** |
| **Chicken_XM_040697196.2** | **11** | **10** | **1395** | **628** | **596** |  | **146** | **174** | **173** | **135** | **82** | **84** | **163** | **113** | **215** | **484** | **342** |
| **Turkey_XM_003204499.4** | **11** | **10** | **1395** | **142** | **107** |  | **149** | **174** | **173** | **135** | **82** | **84** | **163** | **113** | **215** | **281** | **139** |
| **Japanese Quail_XM_015859458.2** | **11** | **10** | **1395** | **256** | **224** |  | **146** | **174** | **173** | **135** | **82** | **84** | **163** | **113** | **215** | **490** | **348** |
| **Lesser Prairie Chicken_XM_052685144.1** | **11** | **10** | **1395** | **192** | **160** |  | **146** | **174** | **173** | **135** | **82** | **84** | **163** | **113** | **215** | **483** | **341** |
| **Ring-necked Pheasant_XM_031610929.1** | **11** | **10** | **1395** | **192** | **160** |  | **146** | **174** | **173** | **135** | **82** | **84** | **163** | **113** | **215** | **164** | **22** |
| **Greater Sage-grouse_XM_042825909.1** | **11** | **10** | **1395** | **104** | **72** |  | **146** | **174** | **173** | **135** | **82** | **84** | **163** | **113** | **215** | **483** | **340** |
| **Rock Ptarmigan_XM_048937597.1** | **12** | **11** | **1395** | **261** | **229** |  | **146** | **174** | **173** | **135** | **82** | **84** | **163** | **113** | **215** | **646** | **504** |
| **White-tailed Ptarmigan_XM_042893635.1** | **12** | **11** | **1395** | **108** | **76** |  | **146** | **174** | **173** | **135** | **82** | **84** | **163** | **113** | **215** | **483** | **341** |
| **Helmeted guineafowl_XM_021390001.1** | **11** | **10** | **1395** | **195** | **163** |  | **146** | **174** | **173** | **135** | **82** | **84** | **163** | **113** | **215** | **475** | **333** |
| **Swan Goose_XM_048071323.2** | **10** | **10** | **1383** | **286** | **239** |  | **149** | **174** | **173** | **135** | **82** | **84** | **163** | **113** | **215** | **477** | **335** |
| **Mute Swan_XM_040552629.1** | **11** | **10** | **1383** | **361** | **314** |  | **149** | **174** | **173** | **135** | **82** | **84** | **163** | **113** | **215** | **478** | **336** |
| **Mallard_XM_027455225.3** | **11** | **10** | **1383** | **252** | **205** |  | **149** | **174** | **173** | **135** | **82** | **84** | **163** | **113** | **215** | **475** | **333** |
| **Tufted Duck_XM_035320944.1** | **11** | **10** | **1383** | **99** | **52** |  | **149** | **174** | **173** | **135** | **82** | **84** | **163** | **113** | **215** | **484** | **342** |
| **Mouse_NM_001409623.1** | **12** | **11** | **1386** | **375** | **532** | **98** | **118** | **174** | **173** | **135** | **82** | **84** | **163** | **113** | **215** | **896** | **751** |
| **Human_NM_001287188.2** | **12** | **11** | **1386** | **621** | **207** | **110** | **119** | **174** | **173** | **135** | **82** | **84** | **163** | **113** | **215** | **901** | **756** |
| **Human_NM_002539.3** | **12** | **11** | **1386** | **334** | **207** | **110** | **119** | **174** | **173** | **135** | **82** | **84** | **163** | **113** | **215** | **901** | **756** |

**Supplementary Table S5.** Potential functional modification sites of the Wuding chicken ODC protein.

| **Name of modification site** | **Serial no.** | **Location and amino composition** |
| --- | --- | --- |
| Casein kinase II phosphorylation site | PS00006 | 9–12: SkeE; 16–19: TflD; 23–26: TakD 36–39: SssD; 37–40：SsdD; 95–98: SktE; 139–142: SevE; 160–163: TtdD; 207–210: TdpE; 305–308: TgsD; 307–310: SddE; 449–452: SgiE |
| Protein kinase C phosphorylation site | PS00005 | 23–25: TaK; 171–173: SvK; 177–179: TlK; 180–182: TsR |
| N-myristoylation site | PS00008 | 3–8: GImgSF; 203–208: GSgcTD; 226–231: GAelGF; 240–245: GGfpGS; 325–330: GVygSF; 328–333: GSfnCI; 353–358: GCysCS |
| N-glycosylation site | PS00001 | 75–78: NDSE |

**Supplementary Table S6.** Proportions of amino acids forming the secondary structure of ODC proteins across different species.

| **species** | **alpha helix** | **extended strand** | **beta turn** | **random coil** |
| --- | --- | --- | --- | --- |
| WuDing chicken | 36.85% | 16.16% | 6.25% | 40.73% |
| Chicekn_NP_001161238.2 | 37.28% | 17.03% | 6.03% | 39.66% |
| Japanese_quail_XP_015714944.1 | 36.85% | 16.38% | 5.39% | 41.38% |
| Turkey_XP_003204547.1 | 35.13% | 17.89% | 6.47% | 40.52% |
| Ring-necked_pheasant_XP_031466790.1 | 35.78% | 17.67% | 7.11% | 39.44% |
| Lesser prairie-chicken_XP_052541104.1 | 36.64% | 16.81% | 6.68% | 39.87% |

**Supplementary Table S7.** Physicochemical properties of ODC proteins in Wuding chickens and other species.

| **Physicochemical  characteristics** | **1** | **2** | **3** | **4** | **5** | **6** | **7** | **8** | **9** | **10** |
| --- | --- | --- | --- | --- | --- | --- | --- | --- | --- | --- |
| Number of amino acids | 464 | 464 | 464 | 464 | 464 | 464 | 464 | 460 | 460 | 460 |
| Molecular weight (kDa) | 51.22 | 51.25 | 51.17 | 51.18 | 51.15 | 51.18 | 51.18 | 50.90 | 50.99 | 50.98 |
| Isoelectric point (PI) | 4.93 | 4.93 | 4.89 | 4.89 | 4.89 | 4.89 | 4.92 | 5 | 5.05 | 5.04 |
| Negatively charged residues (Asp+Glu) | 65 | 65 | 65 | 65 | 65 | 65 | 64 | 62 | 62.00 | 62 |
| Positively charged residues (Arg+Lys) | 46 | 46 | 45 | 45 | 45 | 45 | 45 | 46 | 46.00 | 46 |
| Hydrophobic amino acids (A, I, L, F, W, V) | 169 | 169 | 170 | 171 | 171 | 171 | 171 | 171 | 171.00 | 171 |
| Polar amino acids (N, C, Q, S, T, Y) | 114 | 115 | 115 | 115 | 115 | 114 | 116 | 116 | 113.00 | 114 |
| Instability index (II) | 44.58 | 45.7 | 45.56 | 46.47 | 46.16 | 46.43 | 45.62 | 43.62 | 41.69 | 43.73 |
| Grand average of hydropathicity (GRAVY) | -0.07 | -0.071 | -0.046 | -0.056 | -0.042 | -0.036 | -0.049 | -0.094 | -0.084 | -0.073 |
| Aliphatic index (AI) | 87 | 87 | 87.41 | 88.04 | 88.25 | 88.25 | 88.25 | 86.3 | 86.72 | 86.31 |

Note: 1, Wuding chicken; 2, Chicken (Gallus gallus); 3, Turkey; 4, Japanese quail; 5, Lesser prairie-chicken; 6, Greater sage-grouse; 7, Helmeted guineafowl; 8, Mallard; 9, Swan goose; 10, Budgerigar (Parrot).

**
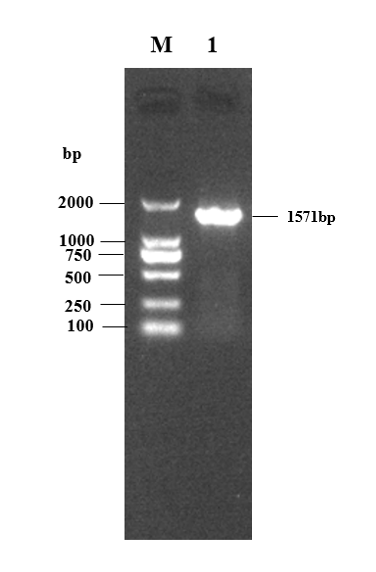
**

**Supplementary Figure S1. PCR amplification products of the ODC gene in Wuding chickens.**

M: DL2000 DNA Marker; 1: PCR amplification product of the Wuding chicken ODC gene.

**
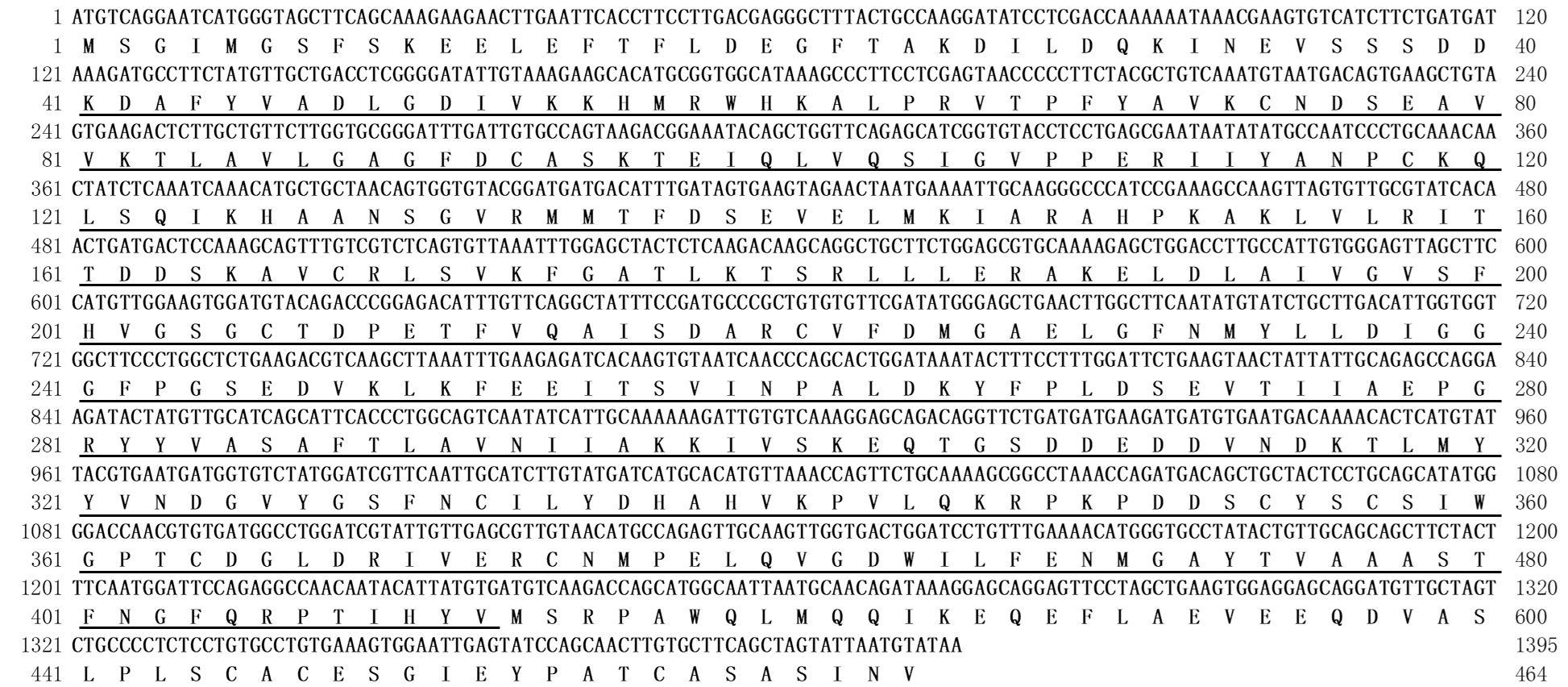
**

**Supplementary Figure S2. The coding sequence (CDS) and encoded amino acid sequence of the Wuding chicken ODC gene obtained in this study.**

Note: ATG indicates the start codon; * indicates the stop codon. The underlined region represents the conserved domain.

**Supplementary Figure S3. Homology and divergence of ODC amino acid sequences among Phasianidae and non-Phasianidae species.**

Note: Values above the diagonal represent sequence identity (homology), and values below the diagonal represent the degree of sequence divergence.

**
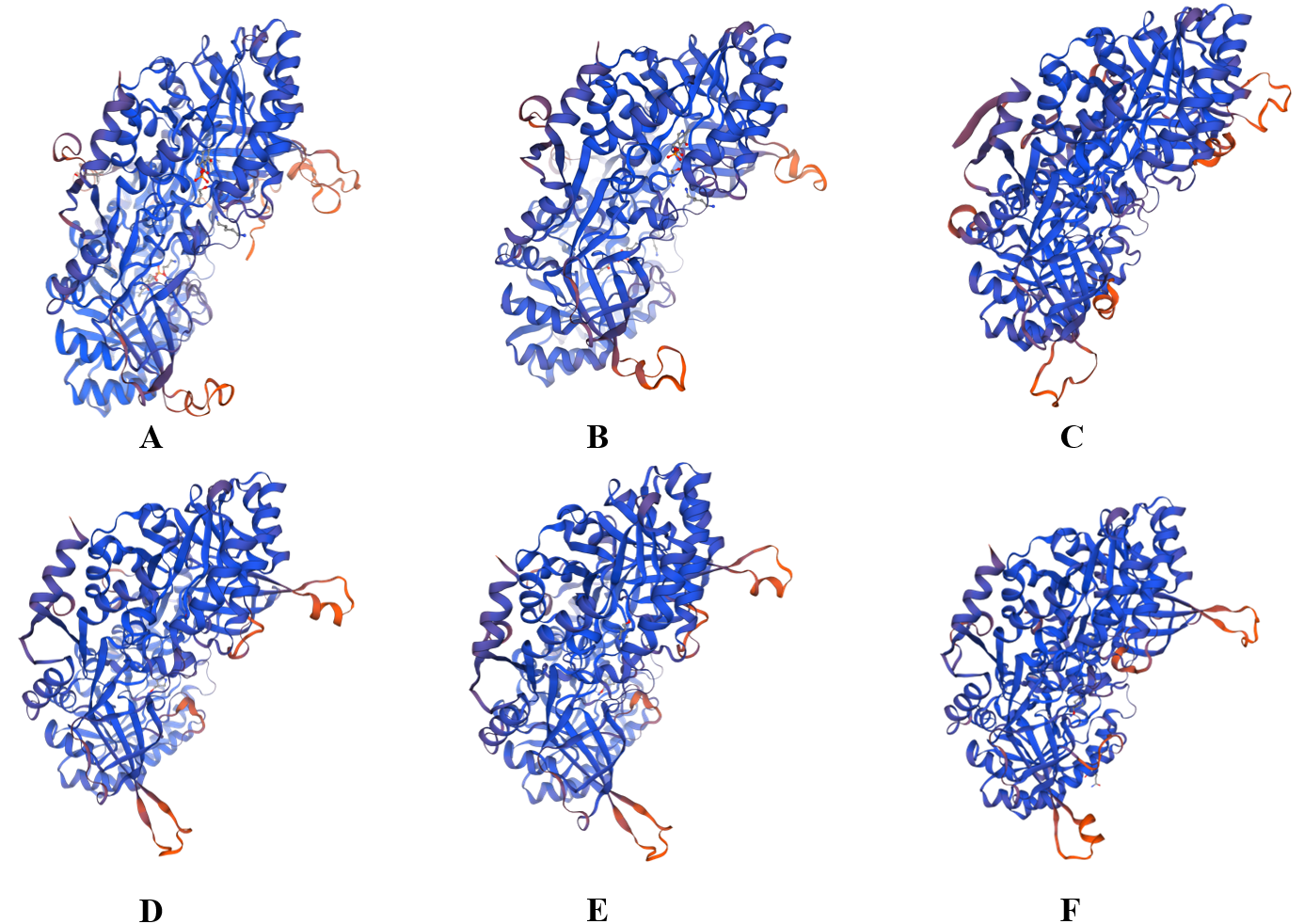
**

**Supplementary Figure S4. Predicted tertiary structures of ODC proteins in Wuding chickens and other Phasianidae species.**

(A) Wuding Chicken ODC; (B) Chicken (NP_001161238.2); (C) Japanese quail (XP_015714944.1); (D) Turkey (XP_003204547.1); (E) Ring-necked pheasant (XP_031466790.1); (F) Lesser prairie-chicken (XP_052541104.1).
